# A Multi-Feature Fusion Slam System Attaching Semantic Invariant to Points and Lines

**DOI:** 10.3390/s21041196

**Published:** 2021-02-08

**Authors:** Gang Li, Yawen Zeng, Huilan Huang, Shaojian Song, Bin Liu, Xiang Liao

**Affiliations:** 1 College of Electrical Engineering, Guangxi University, Nanning 530000, China; ligangac@gxu.edu.cn (G.L.); 1812302024@st.gxu.edu.cn (Y.Z.); ssjlb@gxu.edu.cn (S.S.); bingo.liu@csu.edu.cn (B.L.); 1812302016@st.gxu.edu.cn (X.L.); 2 College of Mechanical Engineering, Guangxi University, Nanning 530000, China; 3 College of Automation, Central South University, Changsha 410083, China

**Keywords:** visual SLAM, point and line features, semantic segmentation, LSD feature extraction, reprojection error

## Abstract

The traditional simultaneous localization and mapping (SLAM) system uses static points of the environment as features for real-time localization and mapping. When there are few available point features, the system is difficult to implement. A feasible solution is to introduce line features. In complex scenarios containing rich line segments, the description of line segments is not strongly differentiated, which can lead to incorrect association of line segment data, thus introducing errors into the system and aggravating the cumulative error of the system. To address this problem, a point-line stereo visual SLAM system incorporating semantic invariants is proposed in this paper. This system improves the accuracy of line feature matching by fusing line features with image semantic invariant information. When defining the error function, the semantic invariant is fused with the reprojection error function, and the semantic constraint is applied to reduce the cumulative error of the poses in the long-term tracking process. Experiments on the Office sequence of the TartanAir dataset and the KITTI dataset show that this system improves the matching accuracy of line features and suppresses the cumulative error of the SLAM system to some extent, and the mean relative pose error (RPE) is 1.38 and 0.0593 m, respectively.

## 1. Introduction

Since the introduction of Industry 4.0, the robot-led intelligent manufacturing industry has become the backbone of industrial development. The visual simultaneous localization and mapping (SLAM) [1] system is the core component that allows robots to explore unknown environments to self-localize and build maps. Visual SLAM relies on inexpensive lightweight cameras that can effectively sense the appearance of the environment, making the SLAM system, which relies only on vision sensors, a hot issue in the field of robotics. The framework of the visual SLAM system is maturing. Although, the research field of visual SLAM has made great progress [2,3,4,5,6,7,8,9,10,11]. However, the variability of the real environment makes the accuracy of data association unreliable or even invalid. This leads to a reduction in the robustness of the system and makes it difficult to meet realistic requirements. Therefore, how to improve the robustness of data association is important to reduce the cumulative error of visual SLAM and improve the system’s overall robustness.

Visual SLAM systems are classified based on the employed tracking method into direct tracking-based and indirect tracking-based methods. Direct tracking-based methods, such as large-scale direct monocular SLAM (LSD-SLAM) [5], direct sparse odometry (DSO) [6], and semi-direct monocular visual odometry (SVO) [7], perform estimation of the pose based on minimizing the photometric projection error. These methods are sensitive to illumination transformations and have poor differentiation between individual pixels. In contrast, the indirect tracking-based method estimates a camera pose by tracking point features of the image. Representative algorithms are parallel tracking and mapping (PTAM) [8], ORB-SLAM2 [9], RGBD SLAM-v2 [10], etc. Point features are insensitive to illumination interference and easy to extract in textured scenes. However, extraction is difficult in scenes with a low-texture environment or motion blur. The robustness of the system is affected, which can lead to failure in severe cases. There are a large number of line features in the real environment that have the same characteristics of invariant illumination and viewpoint as point features and are easy to extract [12]. Hence, the interference caused by low-texture scenes can be overcome, and the complete information about the environment structure can be reflected. Therefore, the SLAM system involving tracking line features was born [13,14,15]. Line features are sensitive to occlusion and do not have strong identification in regions with a lack of texture or high repetition; this results in matching failures and less reliable pose solving than SLAM systems relying only on point features. The tracking of line features is extremely time-consuming and cannot meet the real-time requirements of the SLAM system. Therefore, point and line feature fusion has been applied to SLAM systems [16,17,18,19,20,21,22].

To reduce the generation of cumulative errors, the existing solution is to perform local optimization of the poses and reduce the drift of the trajectory by establishing more constraints between multiple frames of the image in the short term. When the constraints fail, the error still accumulates. The other solution is to establish a long-term constraint by adopting a loop closure to correct the cumulative error, but this solution strictly depends on loop closure detection.

**Figure 1:**
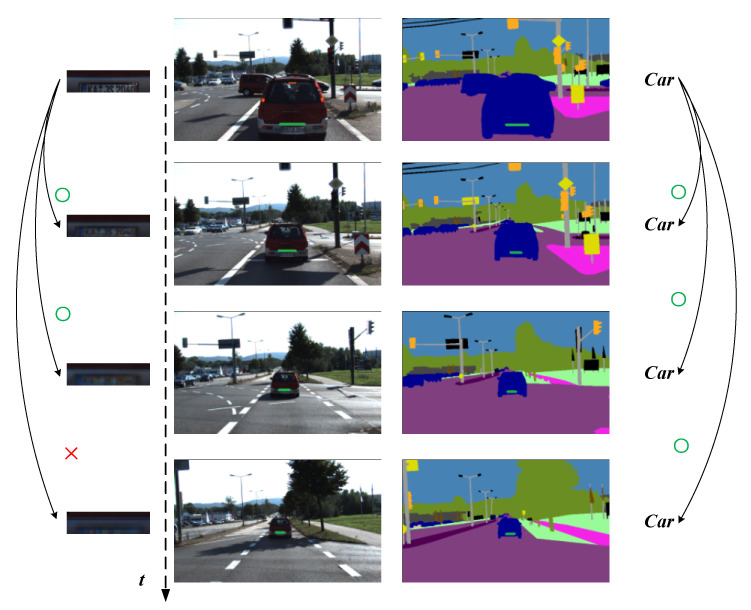
Description of feature semantic invariance. When the car is moving away, the pixels around the line segment change dramatically, but its semantic description remains unchanged.

The rapid development of computer image technologies in recent years, such as deep learning, object detection, and semantic segmentation, provides more possibilities for robots to improve scene understanding. Semantic segmentation [23] is a pixel-level classification technique. Each pixel in an image is classified into a corresponding category; applying semantic segmentation to SLAM systems to improve the robustness of data association is a relatively popular research topic [24,25,26]. In the SLAM system, the movement of the camera over time results in the features changing in viewpoint, scale, and illumination, but not in its semantic description. As shown in Figure 1, when tracking a line segment on a car, the pixels around the line segment change drastically due to the change in distance; this does not match well and leads to tracking failure. However, the semantic description of this line segment belongs to the category of cars, which is not affected by scale and illumination changes. The semantic description of the line segment is then treated as invariant, and the mid-term tracking of line segments is established through the semantic label’s consistency constraint of the line segments and its reprojected features.

At present, the theory development related to line segments is not mature enough, mainly in the lack of accurate description of line segments, which can lead to wrong data association occurring in complicated scenes that include many line segments [27]. This leads to the problem that after the introduction of line segments in SLAM systems based on point-line features, the matching accuracy of line segments is low, which results in the accumulation of system errors.

In this paper, a robust stereo SLAM system with point and line features that combines the semantic invariant is proposed. Specifically, the main contributions of this paper are the following:

- An improved line segment matching method is proposed. We apply the results of semantic segmentation to line segment matching to improve the data association of line segments.
- We define the semantic reprojection error function of line segments and apply it to the pose optimization process to improve the robustness of data association. In this way, the mid-term tracking of line segments is achieved, and the drift problem of trajectories is reduced.

## 2. Related Work

**Figure 2:**
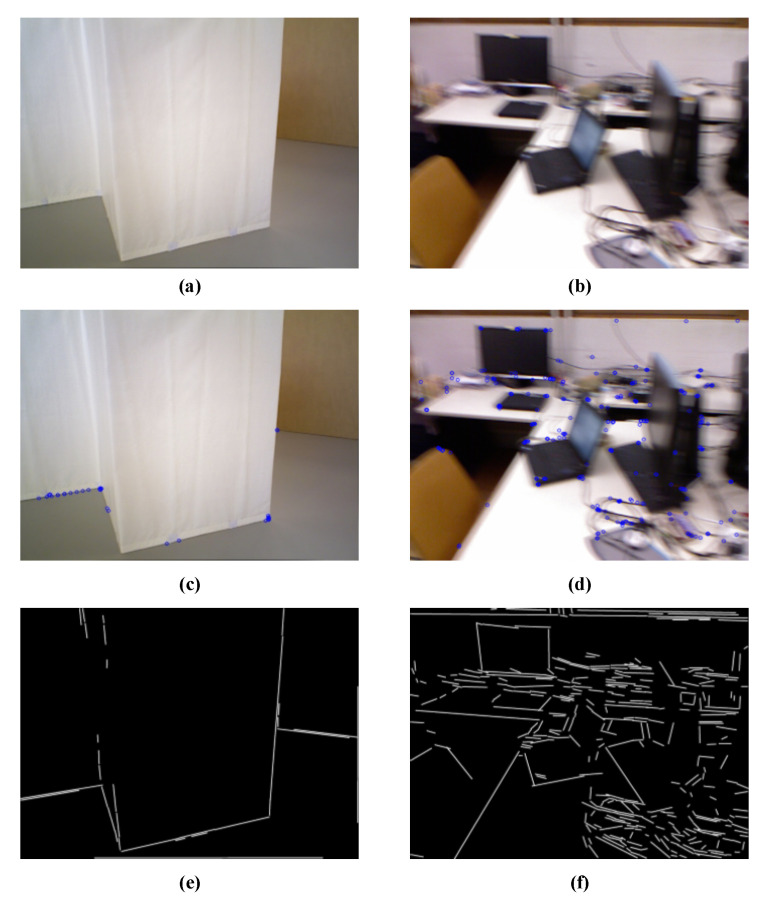
The performance of point and line features in areas of low texture and motion blur. (**a**,**b**) A low-texture scene and motion blur scene, respectively. By comparing the ORB feature points (see figures (**c**,**d**)) and LSD line segments (see figures (**e**,**f**)) extracted from the images, it can be seen that the line segments are more responsive to the environment structure information.

The accuracy of indirect tracking-based SLAM pose estimation relies on the extraction and accurate matching of image features. Point features of images, such as oriented FAST and rotated BRIEF (ORB) [28], speeded-up robust features (SURFs) [29], and scale invariant feature transform (SIFT) [30], are insensitive to illumination changes and easy to extract. Classical visual SLAM systems are designed based on point feature tracking. However, in scenes where the image texture is blurred or missing, the point features might lose the advantage of easy extraction, leading to an insufficient number of feature points and a serious impact on the accuracy of pose estimation, such that the system might even fail. The line segment performs better than the point feature for the same area. As shown in Figure 2, the line segments can reflect the structural information of the environment more completely. Thus, line segments became the technical breakthrough point for SLAM.

In 2006, Smith et al. [13] applied line segments to the extended Kalman filter SLAM (EKF-SLAM) system. A line segment was detected by connecting several adjacent key points to achieve real-time performance. Zhang et al. [14] first proposed a stereo SLAM system based on line segments; this system realized the map construction and loop closure detection function based on line segment tracking. Before 2012, the theoretical development of line segment extraction, description, and matching methods was not complete enough, which resulted in fewer applications of line segments in SLAM systems. After line segment detector (LSD) [31] and line band descriptor (LBD) [32] algorithms were proposed, the extraction and description of line segments became more accurate. Thus, line segments became widely used in SLAM systems. However, computing the poses using only line segments is not as reliable as that through the computation of poses based on point features. Xie et al. then proposed a robust efficient visual SLAM system that utilizes heterogeneous point and line features [18]. The LSD algorithm and LBD algorithm are used for the extraction and description of line segments in this system, respectively. In the process of pose optimization, the method of minimizing the reprojection error was used for optimization, and the Jacobian matrix of the line segment reprojection error was derived. This algorithm simply added up the detection results of point and line features when constructing the error function, which introduced matching error of line segments and directly affected the accuracy of data association.

For greater utilization of environmental information, Suleymanov et al. [33] used deep learning to infer the boundaries of occluded roads to improve the localization accuracy of their system. Semantic SLAM supplements SLAM systems with semantic information for environmental understanding. As a result, semantic segmentation has been proposed to be directly applied to data association in SLAM systems with the aim of reducing the generation of cumulative errors. Bowman [25] proposed to combine an object detection framework with the SLAM system to solve the camera’s poses problem by recognizing objects to assist, but an accurate recognition of objects was needed. Konstantinos-Nektarios et al. [26] proposed a medium-term data association approach, named visual semantic odometry (VSO), that enables medium-term tracking of point features by ensuring the consistency of the semantic labels of the point features, and constructed semantic reprojection error terms.

Based on the stereo point-line SLAM system, the present paper aims at the problem that after the introduction of line segments, the accuracy of data association is directly affected by the mismatching of line segments, which aggravates the cumulative error of the system. An effective improvement approach is proposed. Our approach uses semantic invariants to provide constraints for line segments matching to reduce the generation of line feature mismatching. Furthermore, the semantic reprojection error function of the line segment is defined to realize the mid-term tracking of line segments, which effectively reduces the drift of trajectories and improves the robustness of the system.

## 3. System Overview

**Figure 3:**
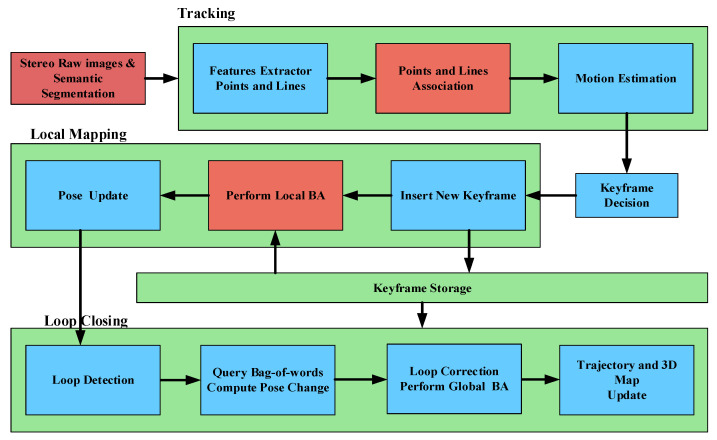
System overview. Our system pipeline is an extension of the ORB-SLAM2 [9]. In the figure, the red squares are the main improved modules in our approach. Our system is composed of three main threads: Tracking, Local Mapping, and Loop Closing. The tracking thread performs pose estimation by data association of point and line features. The local mapping thread adds the new keyframe into the map and optimizes it with BA. The loop closing thread constantly checks for loops and corrects them.

In this section, a brief description of the system design is presented. We indicate in which part of the SLAM system the semantic invariants are mainly applied. The general structure of the proposed system is depicted in Figure 3. The system follows the framework of ORB-SLAM2 [9], and the whole SLAM task runs in parallel according to three threads: visual odometry, local mapping, and loop closure.

The visual odometry part includes feature extraction, matching, and pose estimation. We used the methods described in [9,18] to estimate the poses by processing the point and line features. First, we extract the point and line features in the current frame, and associate the features with those of the previous frame. Based on the results of data associations, a relative motion matrix Δ*T* is calculated. The pose of the current frame is calculated by *T_ew_* = Δ*T·T_rw_*, where *T_ew_* represent the current frame pose, and *T_rw_* represent the previous frame pose.

The local mapping is composed of 3-D landmarks (both points and line segments) and a set of keyframes. If the current frame is determined to be a keyframe, we insert it into the local map to be maintained. The optimization process of the poses is performed by minimizing the sum of the reprojection error term with joint semantic invariants of the reprojection error term.

Loop closure is a process of re-identification and re-localization. The generation of loop closure depends on the similarity of the images. We follow the approach in ORB-SLAM2 [9] and PL-SLAM [17] to determine the similarity of images by computing the similarity of the word vector in the bag-of-words (BoW) [34] approach. Once the loop closure is generated, the global bundle adjustment (BA) process is used to optimize the poses and obtain a globally consistent map.

**Figure 4:**
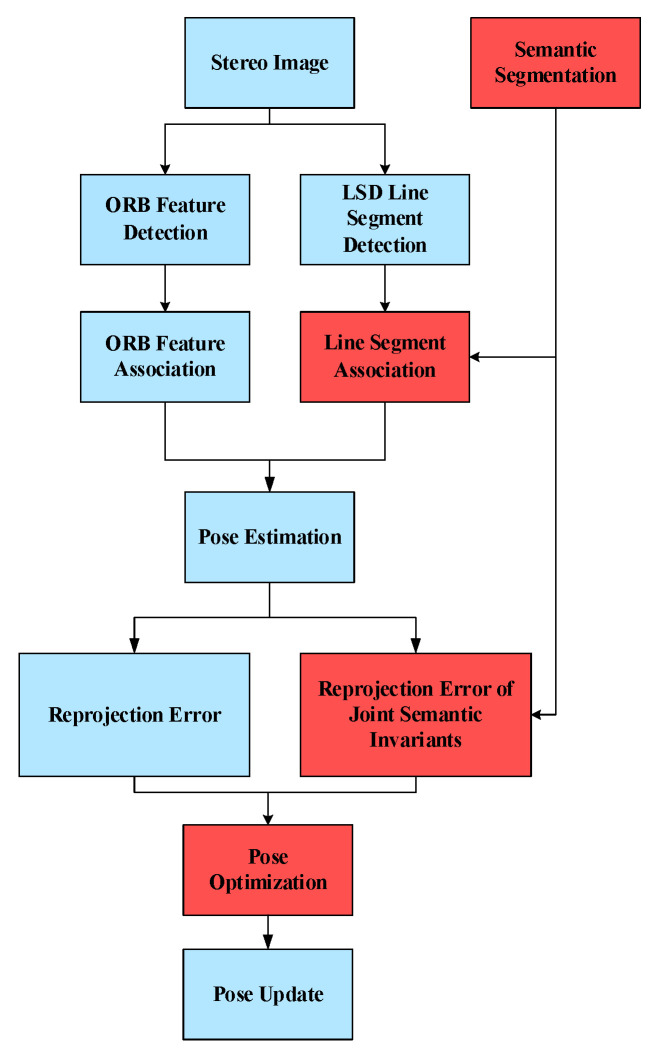
SLAM process incorporating semantic invariants. The red square represents the effective area of the semantic segmentation result. We use semantic invariants as conditional constraint for the data association between line features, and define an error function fused with semantic invariants to optimize the pose.

In this paper, the results of semantic segmentation are mainly applied to the visual odometry and local pose optimization. As shown in Figure 4, the system receives the image sequence and then performs the extraction and matching of point and line features. Since the extraction and matching methods for point features are more complete than line segments, semantic segmentation results are only applied to the association of line segments. Based on existing association methods for line segments, semantic classification of line segments can be done by using the results of semantic segmentation. This provides semantic invariant constraints on the association of line segments and reduces incorrect data associations. When the association results of point and line features are obtained, the landmarks (both points and line segments) in the local map are projected into the current frame and its corresponding semantic segmentation image, respectively. Pose optimization is subsequently performed by minimizing the sum of the reprojection error term with joint semantic invariants of the reprojection error term. Our approach is described in detail in Section 4.

## 4. Semantic Invariants in Line Segment Association and Pose Optimization

In this section, we first introduce the details of the pre-processing of the line segments extracted by the LSD algorithm and the way to apply the results of the semantic segmentation to constrain the data association of the line segments. The problem of how to perform the pose optimization after establishing the medium-term data association about point and line features by semantic invariants is described in Section 4.2.

### 4.1. Pre-Processing and Association of Line Segments

Line segments are extracted using the LSD algorithm. The LSD algorithm is a local straight line detection algorithm that can quickly extract local straight contours in an image without adjusting parameters. However, the line segments are broken into several straight lines due to occlusion or partial blurring, etc. To solve this problem, we follow the method in the literature [18] to merge the broken line segments. Whether a broken line segment satisfies the condition of merging is determined by both the distance between the endpoints and the distance between the line segments. We remove the line segments that do not meet the length threshold after merging.

When the pre-processing is complete, our approach performs semantic classification of the line segments. As shown in the right image of Figure 1, fields of different colors indicate different semantic categories. If an extracted line segment is within a particular color block, the corresponding semantic category label will be given. The following principles are applied to determine whether a line segment belongs to a semantic category:

1. The length of the detected line segment in the category region is greater than the parameter set as threshold D.
2. If the detected line segment lies on the boundary of several semantic categories, it is marked as the category with the highest probability.

**Figure 5:**
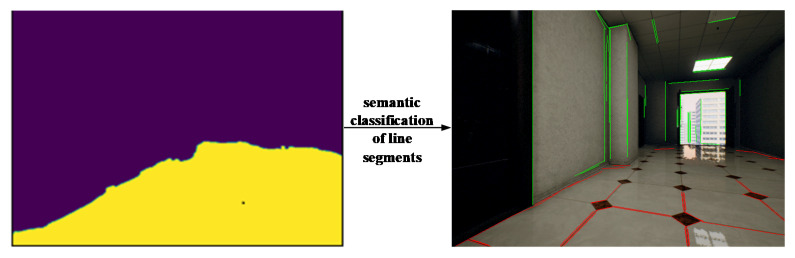
Line segment classification.

Detectron2 is used to predict semantic segmentation of the image. The prediction is composed of ground (yellow area) and non-ground (purple area). Then, the line segments are classified according to the rules proposed above. The classification results are shown in Figure 5.

The data association of line segments should ensure that the line segments belong to the same semantic class and have a high relevance. The relevance of line segments is determined by the description of the local appearance of the line segments, which is provided by the LBD descriptor.

### 4.2. Fusion of Semantic Invariants for Point and Line Reprojection Error Functions

**Figure 6:**
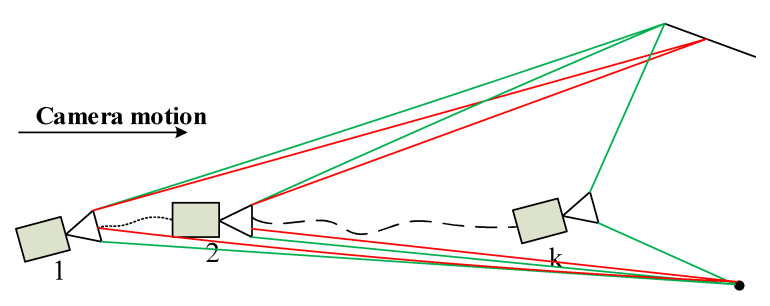
Basic observation and semantic observation of features.

In SLAM systems, there are two main ways to reduce the cumulative error of trajectories. One is to optimize the pose through inter-frame data association to reduce the trajectory drift; this is a short-term constraint. The other one relies on loop closure detection for pose correction, which establishes long-term constraints in the image frame. VSO [26] uses the semantic segmentation information of images to establish a mid-term data association of pairs of points. Line segments also have semantic invariance; therefore, our approach uses this property to establish medium-term data association on line segments. Figure 6 illustrates the data association process for point and line features during camera motion. The red lines indicate the appearance-based constraints on features in the visual odometry framework, and the green line indicates the semantic-based constraints. Camera 1 and camera 2 can establish appearance-based constraints and semantic-based constraints on features. During camera movement, because the description of the feature appearance changes drastically, only the semantic constraint of the feature can be observed in the *k*-th camera. Such semantic constraints can provide a longer-term constraint for feature data association than appearance-based constraints; this is called mid-term tracking of features.

We define an error function by combining semantic invariant with reprojection error:

(1) $$E=E_{base}+E_{sem}$$

where $E_{base}$ is the reprojection error, and $E_{sem}$ is the error function of the fused semantic invariants. By minimizing the error function, the mid-term tracking both of the point and line features is realized, and the drift of the trajectory is reduced.

#### 4.2.1. Definition of $E_{base}$

The point-line feature-based stereo SLAM system usually performs local pose optimization by minimizing the reprojection error [35], given input images $I={\left\{I\right\}}_{k=1}^{k}$, corresponding poses $T={\left\{T\right\}}_{k=1}^{k}$, 3-D points $P_{i}^{N}$, and 3-D line segments $L_{j}^{M}$. The reprojection error function $E_{base}$ is defined as follows:

(2) $$E_{base}=E_{P}+E_{L}$$

where $E_{P}$ and $E_{L}$ represent the reprojection errors of point features and line segments, respectively.

$E_{P}$ is the distance between the observation $\mu_{ik}$ of the *i*-th 3-D point and its reprojection in the *k*-th keyframe:

(3) $$E_{P}=\mu_{ik}-\pi \left(P_{i},K,T_{k}\right)$$

where π(⋅) represents the reprojection coordinates of the 3-D point $P_{i}$; *K* represents the camera’s intrinsic matrix; and $T_{k}$ is the relative motion matrix.

**Figure 7:**
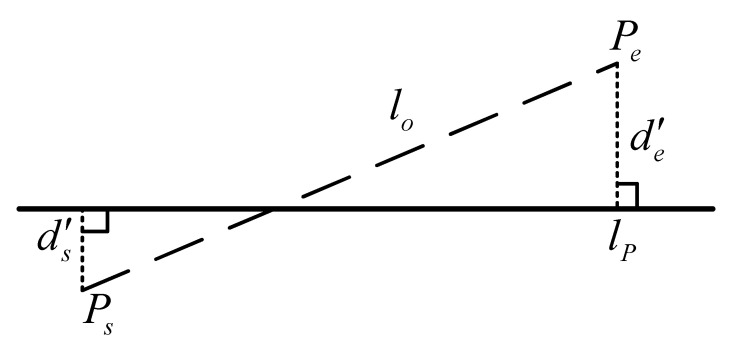
Reprojection error of the line feature.

Uncertainty occurs in the endpoints of line segments in reprojection due to occlusion or other reasons. Therefore, the reprojection error function of the line segment cannot be defined simply by the coordinate’s distance between the observed line and its reprojection. A more precise approach is to use the method in the literature [19], where the reprojection error of the line segment is defined by the sum of the perpendicular distances between the endpoints of the projected line segment and the detected straight line. As shown in Figure 7, $l_{o}$ is the observation of the line segment, and $l_{P}$ is the reprojection of the 3-D line segment; and $d_{s}^{\prime }$ and $d_{e}^{\prime }$ represent the line reprojection errors. Therefore, $E_{L}$ is defined as:

(4) $$E_{L}={d_{s}^{\prime }}^{2}+{d_{e}^{\prime }}^{2}$$

#### 4.2.2. Definition of $E_{sem}$

The error function of the fused semantic invariants describes the probability that the point and line features belong to category C after reprojection. As consistent with the phenomenon elaborated upon in VSO [26], features change drastically during camera motion because of the pixel information around them (see Figure 1). When the camera moves away from the green line, the pixels around the green line have a huge transformation due to the scale shift, which makes the feature fail in tracking. Thus, the constraint of this part of the feature is lost in the data association. In contrast, the semantic description of the feature remains unchanged during the scale change. Therefore, such semantic invariance is applied to data association to establish constraints on features, extend the effective tracking time of features, and reduce the generation of cumulative errors.

For input images $I={\left\{I\right\}}_{k=1}^{k}$, semantic segmentation is performed, and the corresponding semantic segmentation image is $I_{S}={\left\{I_{S}\right\}}_{k=1}^{k}$. Each pixel in $I_{S}$ has a category *C*. Then, for a 3-D point $P_{i}$ projected into $I_{Sk}$, the projection coordinates are $\mu_{i}$, and the projection coordinates have a semantic category $\mu_{i}\in c$, where *c* is a subcategory of *C*. A semantic observation probability model on point features is defined in VSO:

(5) $$P\left(I_{Sk}|T_{k},P_{i},\mu_{i}=c\right)\propto e^{-\frac{1}{2\sigma^{2}}DT_{k}^{C}{\left(\pi \left(P_{i},T_{k}\right)\right)}^{2}}$$

where $DT_{k}^{C}\left(\cdot \right)$ represents the distance from the projection coordinate $\mu_{i}$ to the nearest boundary of the semantic category *C*. *σ* describes the uncertainty of the semantic category *C*. Then, the error function on the fused semantic invariants of the point features can be defined as follows:

(6) $$E_{semP}=\sum_{c\in C}\omega_{i}^{c}\left(-\log\left(P\left(I_{Sk}|T_{k},P_{i},\mu_{i}=c\right)\right)\right)=\sum_{c\in C}\omega_{i}^{c}\cdot \frac{1}{2\sigma^{2}}DT_{k}^{C}{\left(\pi \left(P_{i},T_{k}\right)\right)}^{2}$$

where $\omega_{i}^{c}$ is the category probability vector that describes the case where $P_{i}$ is observed by a series of cameras and the category belongs to *C*. This leads to:

(7) $$\omega_{i}^{c}=\frac{1}{\alpha }\underset{k\in T_{i}}{\Pi }P\left(I_{Sk}|T_{k},P_{i},\mu_{i}=c\right)$$

where α is a constant used to guarantee $\sum_{c\in C}\omega_{i}^{c}=1$.

**Figure 8:**
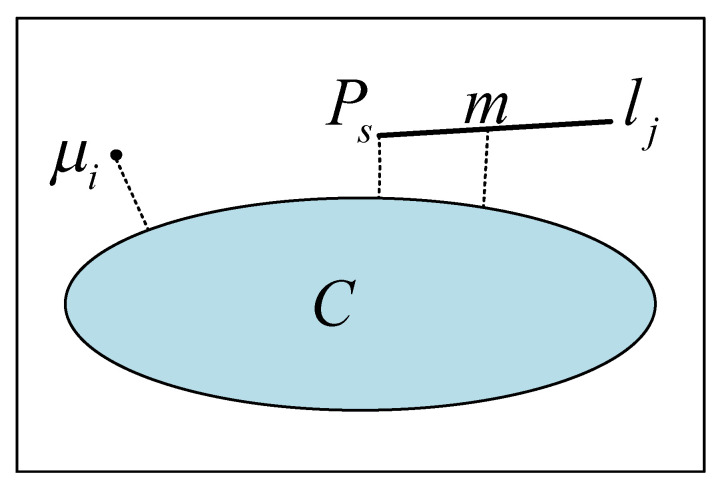
Feature-based semantic observation likelihood. The figure describes the probability that the point and line features reprojected to the semantic segmentation image belong to category *C*. This probability is described by the distance from the point and line features to the semantic boundary. $\mu_{i}$ and $l_{j}$ in the figure represent the point and line features reprojected to the semantic segmentation image; $P_{s}$ and *m* denote the endpoints and midpoints of the line segments, respectively.

Similarly, for a 3-D line $L_{j}$, its projection to $I_{Sk}$ will also make the projected line segment $l_{j}$ have a semantic category $l_{j}\in C$. As shown in Figure 8, the probability of belonging to semantic category *C* for the reprojected line segment $l_{j}$ is described by calculating the two endpoints of the projected line segment and the distance from the midpoint of the line segment to the nearest boundary of semantic category *C*. It can be determined that the smaller the distance $d_{m}$ of the midpoint $P_{m}$ of the line segment from the nearest boundary of *C*, the more likely it is that the line segment belongs to category *C*. To ensure that most of the line segments belong to category *C*, the endpoints with the smallest distance to the nearest boundary of semantic region *C* should also be considered jointly:

(8) $$\{\begin{array}{c} d_{m}=DT_{k}^{C}{\left(\pi \left(P_{mi},T_{k}\right)\right)}^{2}\\ d_{e}=DT_{k}^{C}{\left(\pi \left(P_{ei},T_{k}\right)\right)}^{2} \end{array}$$

where $d_{m}$ and $d_{e}$ represent the distance from the midpoint and the endpoint to the boundary, respectively.

As a result, the probability of a projected line segment belonging to category *C* is described by the distance between the midpoint and endpoints of the projected line segment and the boundary of category *C*. The semantic likelihood model of the line segment is defined as follows:

(9) $$P\left(I_{Sk}/T_{k},L_{j},l_{j}=C\right)\propto e^{-\frac{1}{2\sigma^{2}}\left(DT_{k}^{C}{\left(\pi \left(P_{mi},T_{k}\right)\right)}^{2}+DT_{k}^{C}{\left(\pi \left(P_{ei},T_{k}\right)\right)}^{2}\right)}$$

The error on the fused semantic invariants of the line segments can be defined as:

(10) $$\begin{array}{l} E_{semL}=\sum_{c\in C}\tau_{i}^{c}\left(-\log\left(P\left(I_{Sk}/T_{k},L_{j},l_{j}=C\right)\right)\right)\\ =\sum_{c\in C}\tau_{i}^{c}\cdot \frac{1}{2\sigma^{2}}\left(DT_{k}^{C}{\left(\pi \left(P_{mi},T_{k}\right)\right)}^{2}+DT_{k}^{C}{\left(\pi \left(P_{ei},T_{k}\right)\right)}^{2}\right) \end{array}$$

where $\tau_{i}^{c}$ is the category probability vector describing the case where line segment $L_{j}$ is observed by a series of cameras and the category belongs to *C*:

(11) $$\tau_{i}^{c}=\frac{1}{\beta }\underset{k\in T_{i}}{\Pi }P\left(I_{Sk}/T_{k},L_{j},l_{j}=C\right)$$

The error function of the joint semantic invariants is thus defined as follows:

(12) $$E_{sem}=E_{semP}+E_{semL}$$

The error function for solving the fused semantic invariants follows the EM method in VSO, first solving the category probability vector by E-step keeping the 3-D points and 3D lines unchanged, and M-step keeping the category probability vector unchanged to optimize the camera pose.

## 5. Results

In this section, a series of experiments are performed to verify the effectiveness of the system proposed in this paper. It is necessary to use color images for semantic segmentation. We therefore perform validation using publicly available datasets TartanAir [36] dataset and KITTI [37] dataset, both of which provide color sequences with ground-truth. The TartanAir dataset is an indoor scene dataset, and the KITTI dataset is an outdoor scene dataset. We compare our method with several state-of-the-art methods, including ORB-SLAM2 [9] and PL-SLAM [17]. All experiments are performed on a laptop with Intel i5-4200U CPU, 4GB RAM, and an Ubuntu 16.04 operating system. The semantic segmentation results are obtained using Detectron2, which was introduced by Facebook AI Research [38].

**Figure 9:**
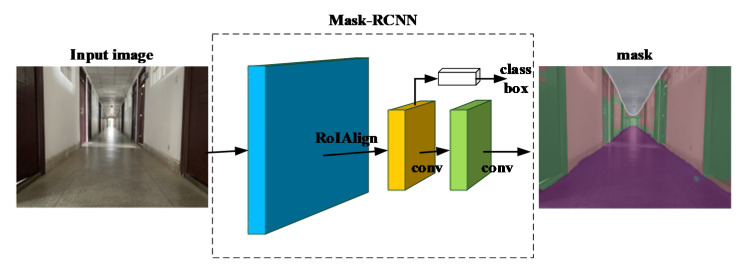
Example of semantic segmentation.

Detectron2 provides a flexible framework based on Mask R-CNN [39], which can add different branches to accomplish tasks, such as object detection, object classification, and semantic segmentation. We use this framework to perform semantic segmentation tasks on the selected sequences, as shown in Figure 9, to prepare for subsequent system operation.

### 5.1. Fusion of Semantic Invariants for Line Feature Matching

**Figure 10:**
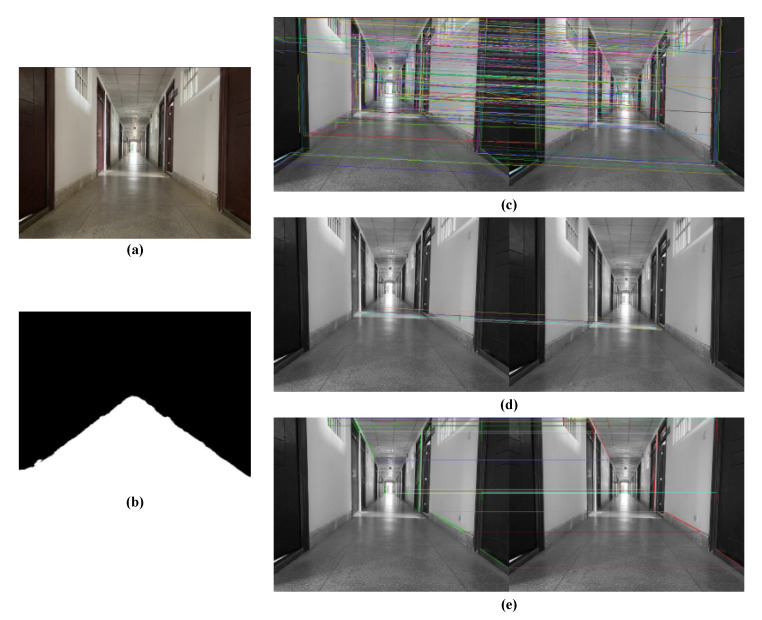
Fusion of semantic invariants for line segment matching. (**a**) The raw image; (**b**) the semantically segmented binary image; (**c**) the results of line segment matching by LBD descriptors in the OpenCV [40] library; (**d**,**e**) line segment matching results after adding semantic invariants.

In this paper, the matching of line segments is constrained by adding semantic invariants to the existing matching method. Two frames in the corridor scene are selected for line feature extraction and matching. Two matching methods are used in the experiments: one is the LBD descriptor matching approach, and the other is our approach. Figure 10 and Table 1 shows the matching results of the two methods.

As can be seen, after adding semantic invariants, the mismatching between line segments is significantly reduced, and the accuracy of line segment matching is improved.

### 5.2. TartanAir Dataset

TartanAir [36] is a dataset with a variable and challenging environment in a virtual scenario. We chose the Office sequence from the TartanAir dataset for our experiments. These sequences contain motion blur and low-texture scenes, and lack dynamic objects. Each sequence contains easy and hard modes. Hard mode means there are drastic illumination changes and camera movements.

We follow two methods to evaluate the performance of the system: absolute trajectory error (ATE), and relative pose error (RPE). The ATE is used to reflect the drift between the ground-truth trajectory and estimated trajectory and is suitable for evaluating the performance of the whole SLAM system. The RPE calculates the difference in the amount of pose change over the same time stamp and is suitable for evaluating the drift of the system.

**Figure 11:**
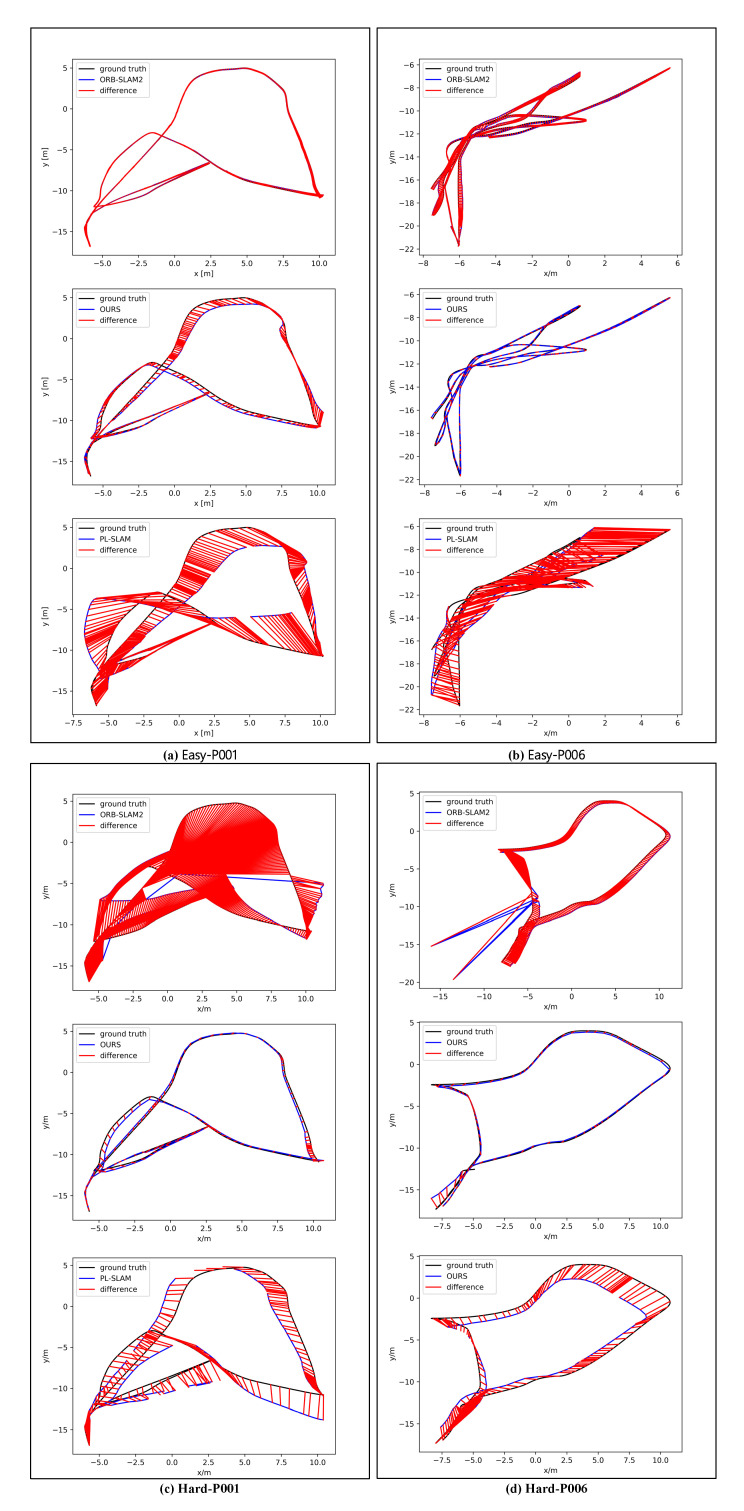
Absolute trajectory errors on the TartanAir dataset. (**a**–**d**) are the ATE comparison of different algorithms in sequences Easy-p001, Easy-P006, Hard-P001 and Hard-P006, respectively. The black line in the figure is the ground-truth, the blue line is the estimated trajectory, and the red area represents the difference between the ground-truth and the estimated trajectory.

Figure 11 shows the ATE for some of the sequences in the TartanAir dataset. We can see the difference between the estimated trajectory and ground-truth of different algorithms. Among the four selected sequences, the system in this paper achieves better results in three of them. In the Easy-P001 sequence, the trajectory estimated by ORB-SLAM2 is closest to the ground-truth, and our method is the next closest. In the Easy-P006, Hard-P001, and Hard-P006 sequences, our approach has excellent performance, and the estimated trajectories are closer to the real trajectories than those of ORB-SLAM2 and PL-SLAM.

**Figure 12:**
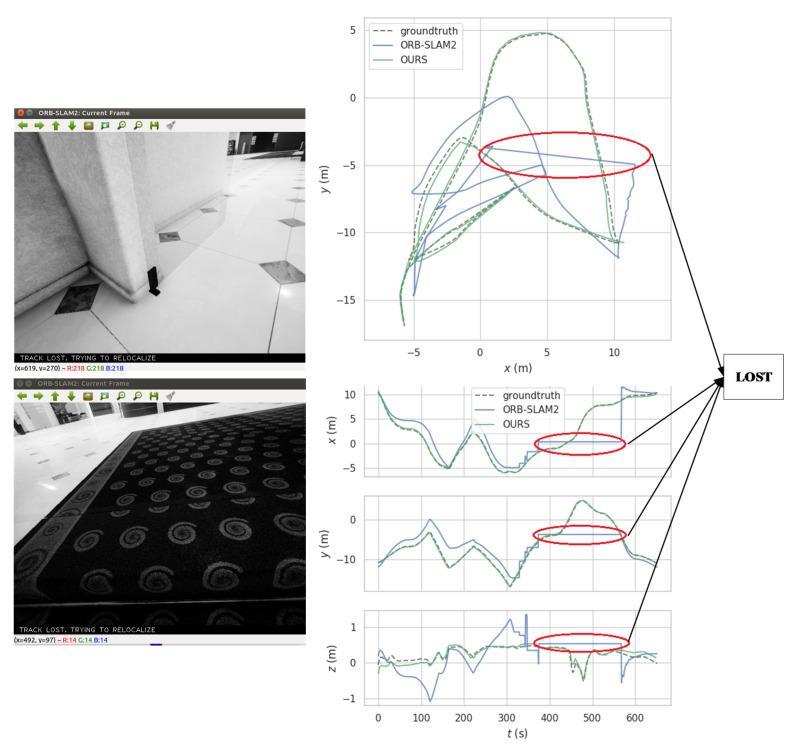
A motion trajectory of ORB-SLAM2 compared with our approach on the Hard-P001 sequence. The red circle in the figure shows that ORB-SLAM2 lost the tracking at runtime, and the left column shows the frames with the tracking lost.

Figure 12 shows the trajectories estimated by ORB-SLAM2 and our approach on the Hard-P001 sequence. We can see that ORB-SLAM2 has tracking loss in this sequence, which occurs in frames 229 and 376–568 of the sequence. In contrast, our approach successfully performed the tracking and estimated a trajectory close to the ground truth.

**Figure 13:**
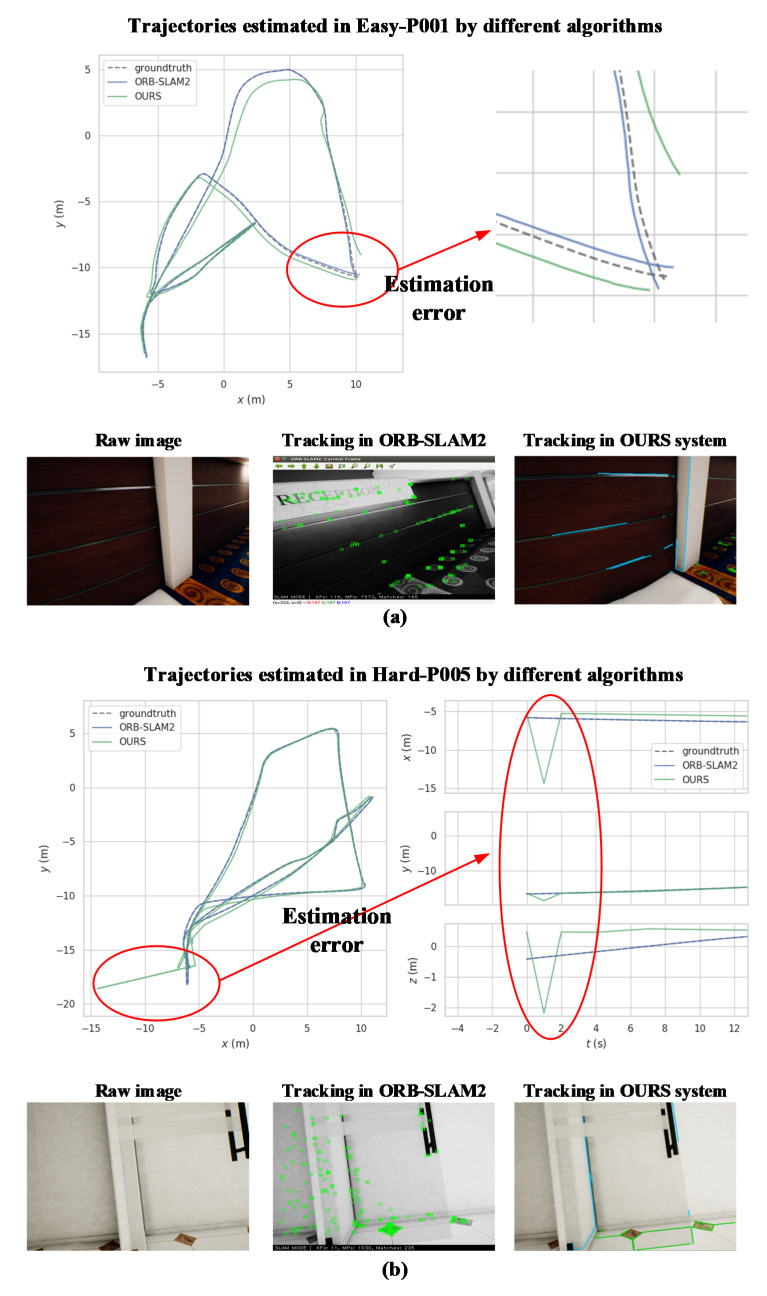
A motion trajectory of ORB-SLAM2 compared with our approach on Easy-P001 and Har-P005 sequences. (**a**,**b**) show the trajectories estimated by different algorithms in thee sequences Easy-P001 and Hard-P005, respectively. The right side of the trajectory shows a zoomed-in version of the trajectory with red circles (pose estimation error). Below the trajectories, the reasons why our system incurs a pose estimation error are shown.

Figure 13 shows the reason why our method has a large error in pose estimation in the Easy-P001 and Hard-P005 sequences. It can be seen that within some image frames, our method cannot extract enough features (both points and lines) for pose estimation. However, ORB-SLAM2 can track smoothly in the same frames and estimate a more accurate pose.

**Figure 14:**
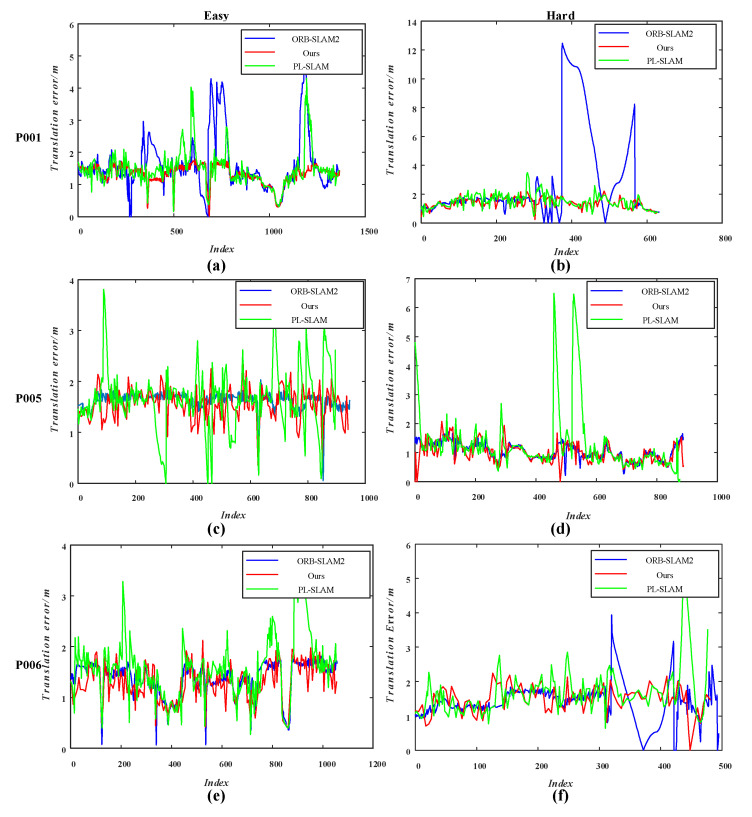
Mean relative pose error of translation on the TartanAir dataset. (**a**,**b**) are the *t_rel_* of different algorithms in Easy-P001, Hard-P001 sequences; (**c**,**d**) are the *t_rel_* of different algorithms in Easy-P005, Hard-P005 sequences; (**e**,**f**) are the *t_rel_* of different algorithms in Easy-P006, Hard-P006 sequences. The blue line, red line, and green line represent the relative pose error of translation for ORB-SLAM2, our approach, and PL-SLAM, respectively.

To verify whether our approach is effective in reducing the generation of cumulative errors, we selected the RPE for evaluation. After calculating the RPE between the trajectory estimated by the system in this paper and the ground-truth, we compared it with the RPE of ORB-SLAM2 and PL-SLAM. The experimental results recorded in Table 2 and Figure 14 describe the degree of drift of the trajectory.

As shown in Table 2, the mean RPE of our approach in the translation direction in the sequences Hard-P001, Easy-P005, Easy-P006, and Hard-P006 is smaller than that of ORB-SLAM2 and PL-SLAM. Furthermore, the mean RPE of rotation of our approach in Easy-P001, Hard-P001, and Easy-P006 is better than that of ORB-SLAM2 and PL-SLAM.

The RPE values for translation are plotted in Figure 14. In the Easy-P001 sequence, the RPE of translation of our method is more uniform, while ORB-SLAM2 and PL-SLAM both produce large undulations, indicating that they produce a large trajectory drift. In the Hard-P001 sequence, the RPEs of the proposed system are closer to those of PL-SLAM, and ORB-SLAM2 produces a large drift in the results estimated in the last 200 frames of the sequence, with a maximum RPE of 12 m. The performance of our method is closer to that of ORB-SLAM2 in the Easy-P005 sequences, with its RPE fluctuating above and below 1.55 m, with a fluctuation range of 0.5 m; meanwhile, PL-SLAM produces a large drift of up to 4 m. In the Hard-P005 sequence, ORB-SLAM2 performs the best, and the RPEs of our method are closer to ORB-SLAM2; meanwhile, PL-SLAM performs the worst. The RPEs of our approach are smoother than those of ORB-SLAM2 and PL-SLAM in the Easy-P006 and Hard-P006 sequences.

The comparison of the experimental results shows that our approach can suppress the trajectory drift better in indoor scenes where there is no interference from dynamic objects.

### 5.3. KITTI Dataset

The KITTI [37] dataset was used to verify that our approach performs properly in texture-rich outdoor scenes. The KITTI dataset is currently the largest test set of autonomous driving scenarios in the world. It covers urban, rural, highway, and other scenes. In this paper, several typical color sequences from the KITTI dataset are used: 00, 04, 07, and 08. Sequence 00 contains multiple loops, 04 is travel in a straight line, 07 contains only one loop, and 08 is travel for a long distance but without a loop.

Table 3 records the RPE of our method and ORB-SLAM2 on the KITTI dataset. There is no significant accuracy improvement of our method in the textured outdoor scenes compared to ORB-SLAM2. This is due to the fact that in outdoor scenes, there are already enough feature points available for the SLAM system to function properly.

**Figure 15:**
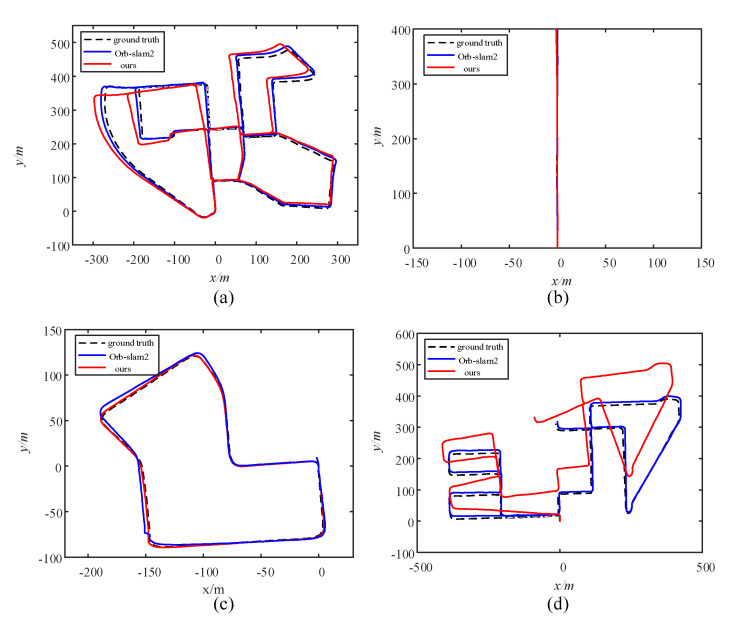
Performance of different algorithms on typical trajectories from the KITTI dataset. (**a**) is the 00 sequence with multiple loops, (**b**) is the 04 sequence with a short straight line, (**c**) is the 07 sequence with one loop, and (**d**) is the 08 sequence with a long line and no loops.

Figure 15 plots the trajectories estimated by different algorithms on the KITTI sequence with the ground-truth provided by the dataset. It can be seen in Figure 15 that in sequences 04 and 07, the accuracy of our approach does not differ much from that of ORB-SLAM2, but in sequences 00 and 08, a large deviation is produced. This is due to the presence of dynamic objects that occupy large areas in the image of sequences 00 and 08.

The experimental results illustrate that applying the results of semantic invariance to the SLAM system in outdoor scenes is not necessarily effective in reducing the trajectory drift of the system. The reason for this result may be that the accuracy of semantic segmentation in outdoor scenes is not high enough, the division of semantic categories is not fine enough, and there is influence from dynamic objects.

### 5.4. Timing Results

In order to complete the evaluation of the proposed system, we present in Table 4 the timing results in each part of the system, for each of the tested datasets. It can be seen that our system and PL-SLAM consume more time than ORB-SLAM in the visual ranging threads. This is due to the addition of the extraction and processing part of the line segment in this thread. Secondly, in the local mapping thread, our system takes the most time, mainly due to the addition of the solving and optimization part of the fusion semantic invariant error function to the pose optimization process. In the loop closing part, since the bag-of-words model based on point and line features is used for loop detection, this increases the time consumption of the system to some extent. Note that the three threads are running in parallel. Finally, on the experimental equipment in this paper, the time consumption of the visual odometry part of the KITTI dataset is 108.49ms, which is about 9 frame/s, whereas the time consumption of the visual odometry part of the TartanAir dataset is 43.84 ms, which is about 22 frame/s. Therefore, our system can basically meet the real-time requirements.

## 6. Conclusions

In this paper, a point-line stereo SLAM system incorporating semantic invariants is proposed. Semantic category labels are given to line segments in order to improve the accuracy of line segment data association. The reprojection error function on the line segment is defined by joint semantic invariants to achieve the mid-term tracking of the line segment, which enables the system to obtain better results when performing local optimization, and reduces the generation of cumulative errors in the trajectory. The effectiveness of our method was verified on the TartanAir dataset and KITTI dataset. The experimental results were compared with those of the ORB-SLAM2 and PL-SLAM system. It is concluded that our proposed algorithm is effective in improving the robustness of the system and reducing the drift of the trajectory in most sequences. However, since the semantic segmentation information is pre-processed, there is no direct real-time segmentation of the original image in the system. Therefore, the subsequent application of real-time semantic segmentation will be considered to further improve the integrity of the system.

## Figures and Tables

**Table 1:** Results of using different methods to associate the data of line segments.

	Number of Detected Line Segments	Number of Data Associations	Number of Correct Data Associations
Classical method	203	178	108
Improved method	46	37	37

**Table 2:** Mean relative pose error (RPE) in the TartanAir dataset. Bold numbers represent the best performances.

Sequence		ORB-SLAM2		PL-SLAM		Ours	
		*t_rel_* (m)	*R_rel_* (°)	*t_rel_* (m)	*R_rel_* (°)	*t_rel_* (m)	*R_rel_* (°)
**P001**	**Easy**	**1.33**	16.15	1.63	16.55	1.37	**16.07**
	**Hard**	1.68	8.73	1.53	7.65	**1.40**	**7.62**
**P005**	**Easy**	1.63	**13.24**	1.61	18.16	**1.57**	13.47
	**Hard**	**1.04**	**7.13**	1.72	10.35	1.19	9.15
**P006**	**Easy**	1.37	12.17	1.71	13.41	**1.28**	**11.77**
	**Hard**	1.48	5.68	1.86	**4.93**	**1.47**	5.05

**Table 3:** Mean relative pose error (RPE) (cm) on the KITTI dataset. Bold numbers represent the best performances.

Sequence	00	04	07	08
**Ours**	5.223	**2.220**	**4.545**	9.584
**ORB-SLAM2**	**3.020**	2.229	4.805	**4.492**

**Table 4:** Average runtime of each part of the system.

		TartanAir, 640 × 480, 25 fps	KITTI, 1241 × 376, 10 fps
Visual Odometry	ORB-SLAM2	36.09 ms	100.07 ms
	PL-SLAM	46.66 ms	123.11 ms
	OURS	43.84 ms	108.49 ms
Local Mapping	ORB-SLAM2	142.31 ms	239.03 ms
	PL-SLAM	105.91 ms	160.93 ms
	OURS	169.40 ms	253.71 ms
Loop Closing	ORB-SLAM2	4.12 ms	9.36 ms
	PL-SLAM	4.67 ms	24.60 ms
	OURS	4.89 ms	38.61 ms
